# Localization of a Peripheral Residual Cyst: Diagnostic Role of CT Scan

**DOI:** 10.1155/2012/760571

**Published:** 2012-02-02

**Authors:** Anshuman Jamdade, Gopakumar R. Nair, Madhur Kapoor, Neeta Sharma, Arya Kundendu

**Affiliations:** ^1^Department of Oral Medicine and Radiology, Mahatma Gandhi Dental College & Hospital, Tonk Road, Sitapura, Jaipur 302022, Rajasthan, India; ^2^Department of Oral Pathology and Microbiology, Mahatma Gandhi Dental College & Hospital, Sitapura, Jaipur 302022, Rajasthan, India

## Abstract

The term *residual cyst* is used most often for retained radicular cyst from teeth that has been removed. Residual cysts are among most common cysts of the jaws. The location of all odontogenic cysts is usually intraosseous. The peripheral (extraosseous) presentations are rare. The peripheral presentation of residual cyst has never been reported in the literature. In this article, the role of CT in diagnosing an unusual peripheral presentation of a residual cyst is discussed.

## 1. Introduction

The higher frequency of occurrence of cysts in the oro-facial region could be attributed to complex embryology and development of teeth and due to presence of odontogenic epithelial remnants. Radicular and residual cysts are by far the most common cyst comprising about 52.3% to 60% of all jaw cysts [1, 2]. Most radicular cysts (60%) are found in the maxilla, especially around incisors and canines [3]. The residual cyst can develop in a dental granuloma that is left after an extraction. They represent approximately 10% of all odontogenic cysts [4]. The radicular cyst does not recur if surgical removal is thorough. If the cystic sac is badly fragmented, leaving epithelial remnants, or if a periapical granuloma is incompletely removed with epithelial rests remaining, a residual cyst may develop in this area months or even years later. The residual cyst is usually asymptomatic and often discovered on routine radiographic examination of an edentulous area. However, they can cause expansion of cortical plates of the jaws and may invaginate into maxillary antrum or depress inferior alveolar canal. Sometimes become painful in case of secondary infection. Radiographically, it is a well-defined round-to-oval radiolucency with a corticated margin. The internal aspect of the cyst is typically radiolucent. Dystrophic calcification may be present in long-standing cyst, commonly with regressing one [3, 5]. However, an infection makes the cortex less apparent [6]. Unfortunately, very little study was carried out on proportion of regression versus growth of the residual cysts. The growing cyst can cause displacement or resorption of adjacent anatomical structure. However, in case of peripheral presentation of the same, it becomes difficult to diagnose exact location and extension of the lesion by conventional radiography. In maxilla, the peripheral residual cyst is a diagnostic dilemma whether it is originated from the alveolus/or the maxillary antrum. Residual cysts often occur centrally (within bone), mostly in the tooth-bearing region. In our case, on clinical examination, it was appeared as a usual intraosseous residual cyst causing cortical expansion while conventional radiographs did not clearly defined the exact location and origin of the cyst but correct application of CT scan revealed a rare peripheral presentation of residual cyst.

## 2. Case Report

A 30-year-old male patient reported to the Department of Oral Medicine and Radiology, Mahatma Gandhi Dental College & Hospital, Jaipur, India, with a chief complaint of slowly progressing swelling on the right side of the face of 5 months duration. Onset was insidious after extraction of a decayed tooth from the same region. Initially, it was small and soft but gradually it became large and hard in consistency. Occasional mild pain was reported especially on pressure and forward bending of the head. The medical history was noncontributory.

**Figure 1:**
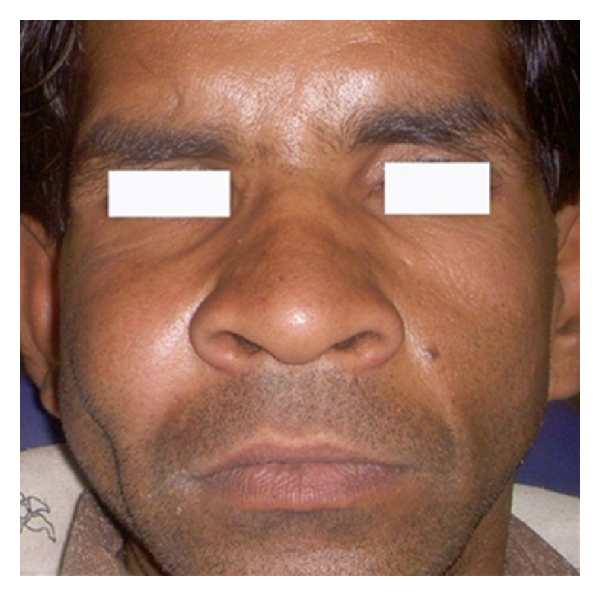
A golf-ball-sized swelling on the right maxilla causing facial asymmetry.

**Figure 2:**
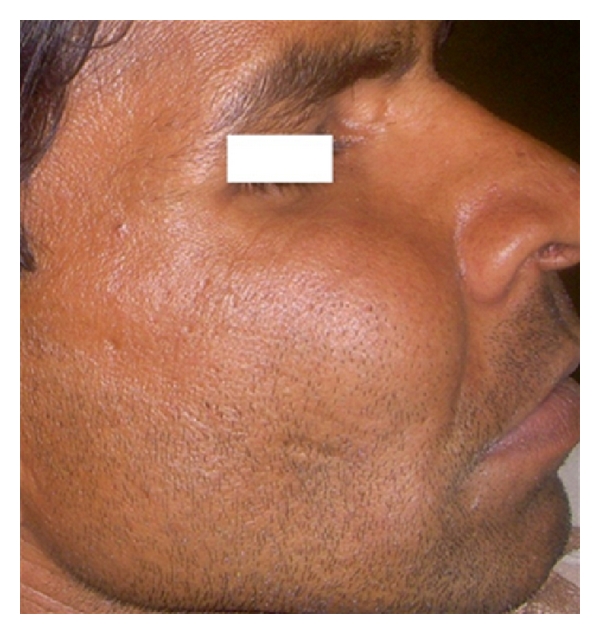
Anteroposterior extension of the swelling.

On examination, patient was a short-statured, average-built, healthy-looking male. A general survey of the patient did not reveal any abnormality of significance. Face showed an obvious asymmetry caused due to a swelling on the right side of the maxilla. The swelling was approximately 3.5 × 3.5 cm. in size, circular in shape, and extending anteroposteriorly from the ala of nose to one cm behind an imaginary vertical line drawn from the lateral canthus of the eye and superoinferiorly, from infraorbital margin to one cm below the ala-tragus line. The color of overlying skin was normal. The swelling was bony hard in consistency, mildly tender on palpation with no change in local temperature. The overlying skin was smooth, intact, and appeared little stretched (Figures 1 and 2). The detailed head and neck examination did not reveal any significant findings.

**Figure 3:**
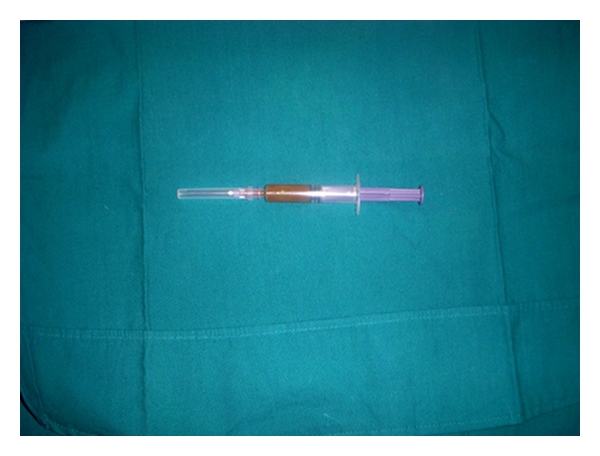
Aspirated material was a red colored, viscous fluid.

 Intraoral examination revealed missing first permanent molar in the right maxillary region with healed extraction socket and normal overlying alveolar mucosa. There was a well-defined, localized swelling obliterating the buccal vestibule of right maxilla. The swelling was extending superoinferiorly 0.5 cm away from gingival margin of premolars and first two molars to upper buccal vestibule and anteroposteriorly from distal of first premolar to distal of second molar. It was dome shaped, soft, fluctuant, and nontender on palpation. The overlying mucosa was smooth, elevated, but of the same color as that of adjacent mucosa and presented with no sign of inflammation. A fine-needle aspiration revealed a dark-red-colored, blood-tinged, and highly viscous fluid. Vitality test gave a positive normal response of the involved teeth (Figure 3).

 After analyzing history and clinical findings, a provisional diagnosis of a residual cyst was arrived at. The following cystic and tumorous lesions were considered as differential diagnosis. These were an odontogenic keratocyst, cystic ameloblastoma, cystic degeneration of adenomatoid odontogenic tumor, and a cyst arising from the maxillary antrum.

**Figure 4:**
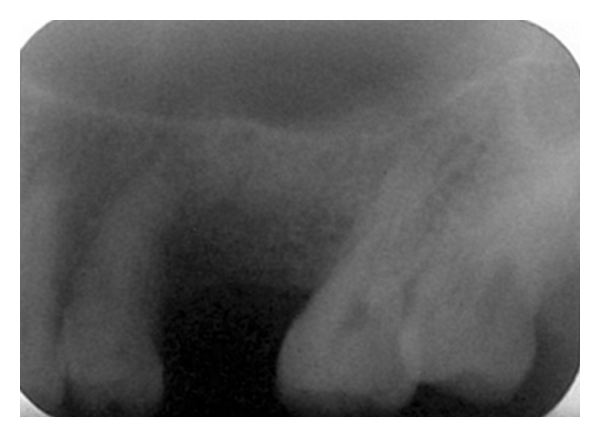
IOPA radiograph of maxillary right posterior region showing a partial radiolucency superimposed on the edentulous region.

**Figure 5:**
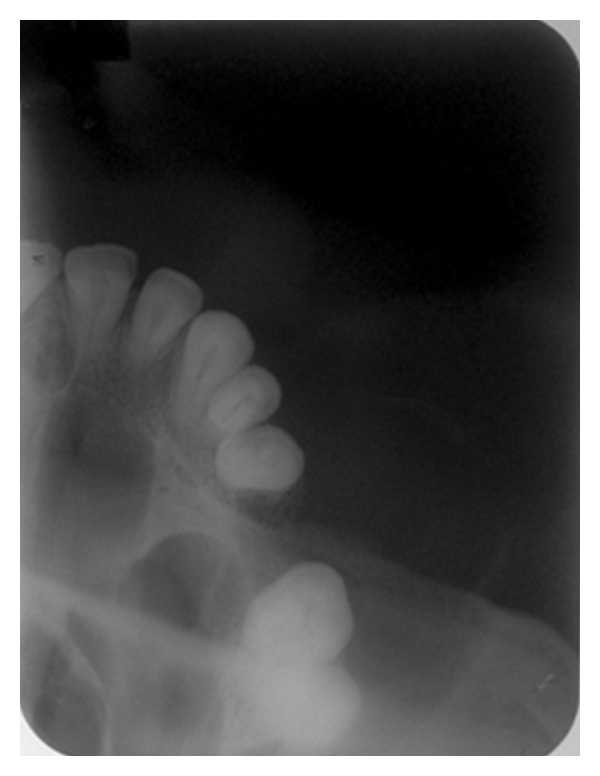
Occlusal view shows a circular radiolucency with a thin discontinuous cortication present on the buccal aspect of the maxilla.

As the swelling was in relation to right maxilla and its alveolar bone, we first carried out intraoral radiographic examinations. Intraoral periapical radiograph of maxillary right posterior teeth region revealed missing first permanent molar with a partial radiolucency superimposed on the edentulous region. There was no evidence of root stump, cystic radiolucency, or any abnormality in relation to the floor of the maxillary sinus (Figure 4). Occlusal radiograph showed a well-defined, unilocular, circular radiolucency present on the buccal aspect of the right maxilla extending from distal aspect of first premolar to distal aspect of third molar surrounded by a thin, discontinuous cortical margin (Figure 5).

**Figure 6:**
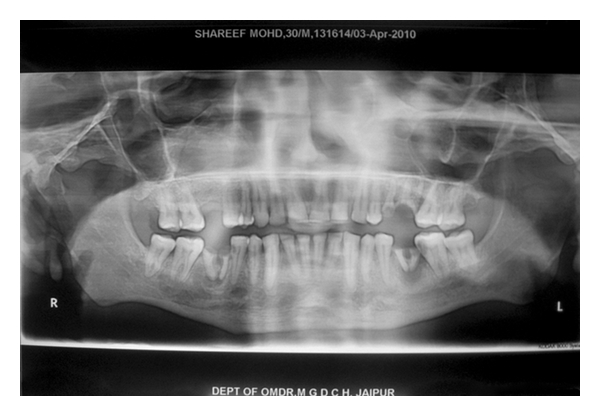
Panoramic view showing a radiolucency with haziness present on the right maxilla, zygomatic arch, anterior wall, and floor of the maxillary sinus were not clearly demarcated.

**Figure 7:**
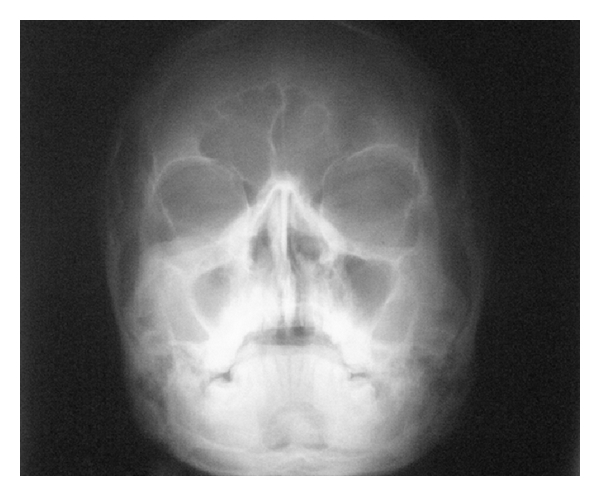
PA Water's view shows haziness with decrease in size of the right maxillary sinus.

 Panoramic radiograph revealed a unilocular radiolucency with haziness surrounded by discontinuous cortication present on the right maxillary antrum region. It was oval in shape, around 3 × 2 cm in size, extending antero-posteriorly from distal of canine to mesial of third molar region. Superoinferiorly, it was not extended to displace teeth or floor of the orbit. The right zygomatic process, anterior wall, and floor of the maxillary sinus were not clearly demarcated indicating lateral, presence of the lesion causing displacement/resorption of these structures. Interestingly, all remaining first permanent molars were grossly decayed and associated with well-defined periapical radiolucencies; probably radicular cysts (Figure 6). Water's view showed a radiopaque haziness with little decrease in the size of the right sinus. No any abnormality detected in relation to medial, lateral, and superior wall of the sinus (Figure 7). 

**Figure 8:**
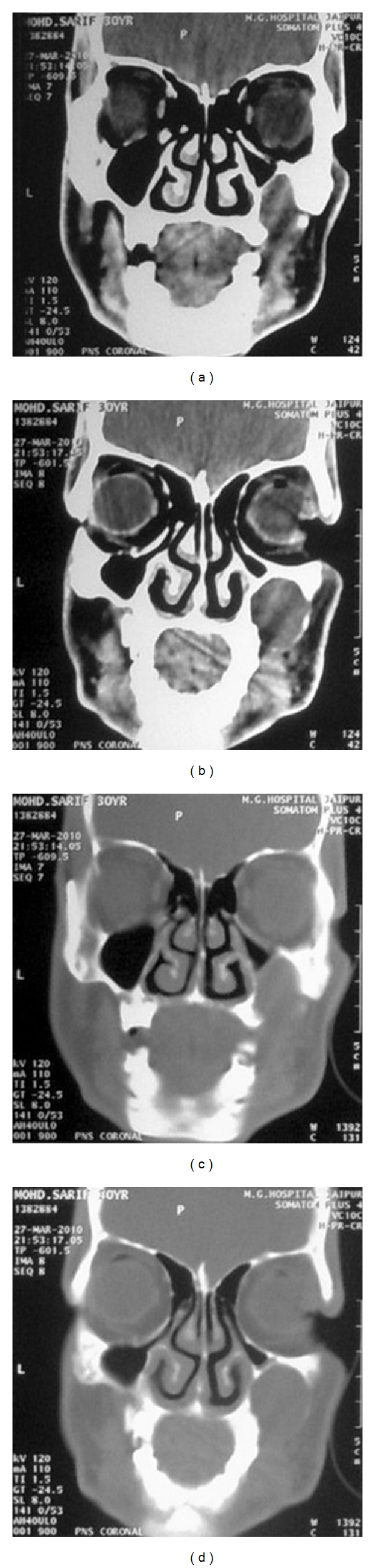
(a, b) CT scan view (coronal section soft tissue window), (c, d) CT scan view (coronal section bone window). Both views show a well defined soft tissue lesion causing displacement of inferolateral wall of the right sinus without involving it.

**Figure 9:**
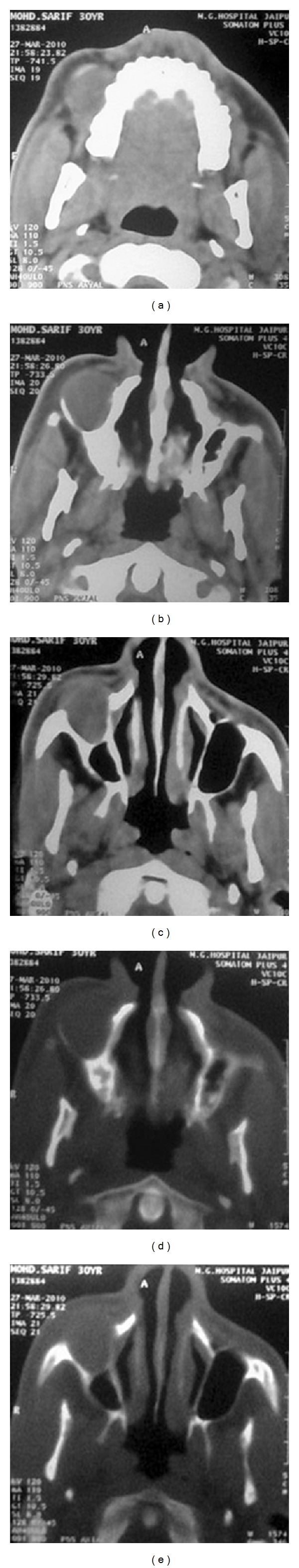
(a, b, c) CT scan view (Axial section soft tissue window), (d, e) CT scan view (Axial section bone window). Both views show a well-defined soft tissue lesion causing displacement of anterolateral wall of the right sinus with involving it.

 CT scan was carried out to rule out the origin of the lesion, sinus, or orbital involvement by the lesion and erosion of the alveolar bone. Coronal section revealed a round soft tissue lesion surrounded by a thin, discontinuous cortication present on the right lateral surface of the maxilla. It is causing pressure and superior displacement of inferiolateral walls of the maxillary sinus, decreasing the size of the sinus without actual perforation or involvement (Figures 8(a)–8(d)). Axial section showed that the lesion is present on the right maxilla pushing anteriolateral wall of the sinus posteriorly decreasing the size of the sinus without actual involvement. There was no any sign of perforation or erosion of respective maxillary bone (Figures 9(a)–9(e)). 

**Figure 10:**
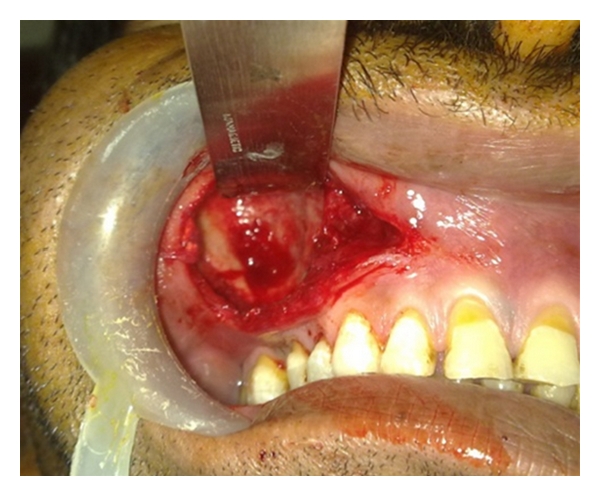
Surgical enucleation of the lesion through an intraoral approach, photograph after removal of the lesion in toto showing bony concavity on anterolateral wall of the sinus.

**Figure 11:**
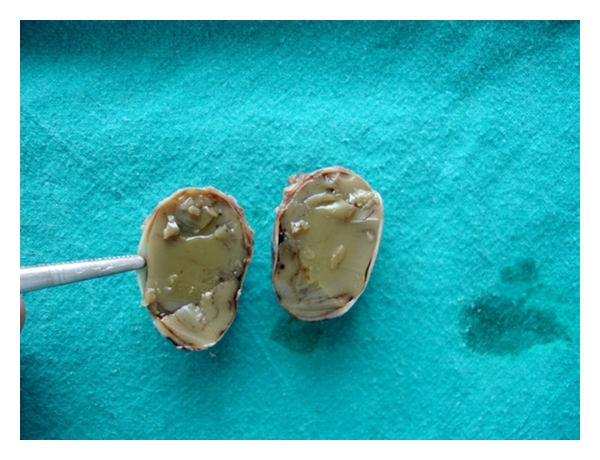
Sectioned gross specimen shows solidified cheese like material surrounded by a soft capsule.

**Figure 12:**
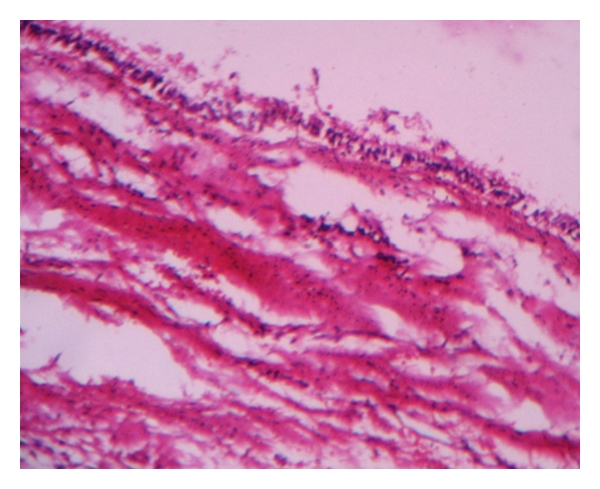
Histopathological section shows features of residual cyst.

 Cytological examination of aspirates was suggestive of chronic inflammatory fluid. The surgical enucleation of the cyst was carried out under local anesthesia and strict asepsis through an intraoral approach (Figure 10). The sectioned gross specimen revealed yellowish, solidified pus like material surrounded by a thin-layered soft capsule (Figure 11). Postsurgical period was uneventful. The histopathological features of atrophic, metaplastic, nonkeratinized, stratified squamous epithelium associated with minimal inflammation confirmed the diagnosis of an established residual cyst (Figure 12). The biochemical analysis of the aspirated fluid revealed increased value of serum globulin and total serum protein with reversed serum albumin/serum globulin ratio (0.5/1). While culture and sensitivity examination of the same revealed that enterobacter species was grown, this was found sensitive to ciprofloxacin, augmentin, azithromycin, doxycycline, and ampicillin-sulbactum combination. These additional investigations of the content supported the diagnosis of an inflammatory cyst.

## 3. Discussion

Residual cyst usually has a typical location and radiographic aspect, allowing an easy diagnosis. These cysts are usually intraosseous with the epicenter is positioned in the former periapical region of an extracted tooth. Occasionally, radicular cysts are present on the lateral aspect of the root in relation to accessory canal of the devitalized tooth. To our knowledge, peripheral (extraosseous) residual cyst has never been reported to date in the English-language literature. Nevertheless, the location does not seem to have any relation to either behavioral or histological features of the cyst [7].

In a study carried out on five hundred ninety four patients with 621 cysts, most of the cysts were inflammatory: 435 cysts (70.1%) and odontogenic: 603 (97%) in their origin [8]. Out of 621 cysts, 112 (18%) were residual, 44 (7.1%) were exacerbated, and 15 (2.4%) recurrent. Residual cysts are more common in the upper jaw (66.2%) and affected predominantly the distal areas of the maxilla—areas adjacent to extracted molars and premolars. The percentage of men with jaw cysts was higher compared to that of women, males to females ratio was 1.22 : 1. The average age of patients with jaw cysts is 35 years. Other authors also report that odontogenic cysts present 90% of all cysts of the jaws [9, 10]. In literature, we found that radicular cysts were the most common cyst type. The male sex is more commonly affected. Cysts affect more frequently the maxilla, and in the maxilla, the alveolar bone is most commonly involved.

CT scan examination revealed a subperiosteal cyst/benign tumor present peripherally on the surface of the maxilla causing superior and posterior displacement of inferiolateral and anterolateral walls of the maxillary sinus, respectively, but not occupying the sinus cavity. In this case, conventional radiographs just revealed a unilocular radiolucency with an incomplete surrounding cortex, present on the surface of the maxilla. However, the origin and location, whether it is intraosseous or extraosseous or from alveolus or maxillary sinus, and its extension and effect on the surrounding structures were not clearly defined. Even histopathological and other additional investigations did not focus much on these aspects of the lesion.

The epithelium of residual cyst originates from the epithelial rests of Malassez, although, in some instances, it arises from the respiratory epithelium of the maxillary sinus when the periapical lesion communicates with the sinus wall. It may also come from oral epithelium from a fistulus tract or oral epithelium proliferating apically from a periodontal pocket. The epithelium may be derived from the surface epithelium or from the epithelium of adjacent glands or hair follicles [11]. Intraneural epithelial islands have been reported in association with a radicular cyst in the mandible and maxilla [12]. In the reported case, we believe that the cystic epithelium either implanted below the periosteum during extraction of the offending tooth or fenestrated the bone. Trauma is always the precipitating factor, and a source of epithelium capable of proliferation at that period of time is required. A chronic inflammatory process would certainly be present when offending tooth is removed and could persist as a result of lysosome or other irritant chemical release from dead or dying cells and at some point trigger an acute inflammatory reaction. If the radicular cyst is inadvertently left behind following tooth extraction, some degree of inflammation may carry on and residual cyst, although to a lesser extend than radicular cysts, have the potential to expand [13].

Radicular cyst arising from deciduous teeth is also one of the possibilities considered. There are cyst-prone individuals who show a particular susceptibility to develop radicular cysts [14]. They have a defective immunological surveillance and suppression mechanism [15]. High and Hirschmann showed that there was an inverse relationship between the percentage of polymorphonuclear leucocytes in the inflammatory infiltrate and cortication of the cyst wall radiographically (*P* < 0.001) [16]. Many radicular cysts are either not detected prior to extraction of an offended tooth or must be left *in situ* following extraction of that tooth. Many residual cysts are slowly resolving especially the asymptomatic one [5, 16]; some undergo renewed growth as the result of cellular breakdown brought about by secondary infection or traumatic irritation and consequent rise in osmotic pressure [14].

No statistically significant differences were found between fluid from apical periodontal and residual cysts on the one hand and autologous serum on the other, or between the different types of cyst fluids [17]. A data from a study suggested that the larger molecules could diffuse across the epithelial linings of cysts. They showed that higher molecular weight proteins are also found in fluids from all types of cyst [18]. Epithelial permeability together with discontinuities in the epithelial lining and intraepithelial channels could allow passage of larger molecules into the cystic luminal fluid. Skaug confirmed that fluid from nonkeratinizing jaw cysts contains high concentrations of protein but supported the view that accumulation of cyst fluid results essentially from inadequate lymphatic drainage of the cyst cavity [17]. Some residual cysts even attended a massive size and caused severe destruction [19]. A case of squamous cell carcinoma arising from a residual cyst in a 55-year-old man is reported [20].

In conclusion, this case report clearly demonstrated the importance of the advanced imaging like CT scan in detecting exact location, extension of the peripheral (extraosseous) residual cyst, and its effect on the adjacent structures especially maxillary sinus. This cyst should be included in the differential diagnosis of peripheral lesions of the jaws.

## References

[1] Shafer WG, Hine MK, Levy BM, Rajendran R, Sivapathasundharam B (2006). *Shafer’s Textbook of Oral Pathology*.

[2] Shear M (1992). *Textbook of Cysts of the Oral Regions*.

[3] White SC, Pharoah MJ (2009). *Textbook of Oral Radiology Principles and Interpretations*.

[4] Main DMG (1970). Epithelial jaw cysts: a clinicopathological reappraisal. *British Journal of Oral Surgery*.

[5] High AS, Hirschmann PN (1986). Age changes in residual radicular cysts. *Journal of Oral Pathology*.

[6] Mortensen H, Winther JE, Birn H (1970). Periapical granulomas and cysts. An investigation of 1,600 cases. *Scandinavian Journal of Dental Research*.

[7] Seyedmajidi M, Feizabadi M (2009). Peripheral calcifying odontogenic cyst. *Archives of Iranian Medicine*.

[8] Pechalova PF, Bakardjiev AG (2009). Cysts of the Jaws: a clinical study of 621 cases. *Acta Stomatologica Croatica*.

[9] Kramer IR, Pindborg JJ, Shear M (1992). *Histological Typing of Odontogenic Tumours*.

[10] Neville BW, Damm DD, Allen CM, Bouquot JE (2002). *Oral & Maxillofacial Pathology*.

[11] Ettinger RL, Manderson RD (1973). Implantation keratinizing epidermoid cysts. A review and case history. *Oral Surgery Oral Medicine and Oral Pathology*.

[12] George DI, Gould AR, Behr MM (1984). Intraneural epithelial islands associated with a periapical cyst. *Oral Surgery, Oral Medicine, Oral Pathology*.

[13] Muglali M, Komerik N, Bulut E, Yarim GF, Celebi N, Sumer M (2008). Cytokine and chemokine levels in radicular and residual cyst fluids. *Journal of Oral Pathology and Medicine*.

[14] Oehlers FAC (1970). Periapical lesions and residual dental cysts. *British Journal of Oral Surgery*.

[15] Toller PA (1970). Protein substances in odontogenic cyst fluids. *British Dental Journal*.

[16] High AS, Hirschmann PN (1988). Symptomatic residual radicular cysts. *Journal of Oral Pathology*.

[17] Skaug N (1973). Proteins in fluid from non keratinizing jaw cysts. 2. Concentrations of total protein, some protein fractions and nitrogen. *Journal of Oral Pathology*.

[18] Smith AJ, Matthews JB, Mason GI, Browne RM (1988). Lactoferrin in aspirates of odontogenic cyst fluid. *Journal of Clinical Pathology*.

[19] Dimitroulis G, Curtin J (1998). Massive residual dental cyst: case report. *Australian Dental Journal*.

[20] Muglali M, Sumer AP (2008). Squamous cell carcinoma arising in a residual cyst: a case report. *Journal of Contemporary Dental Practice*.

